# Long-Term Performance Assessment of Low-Cost Atmospheric Sensors in the Arctic Environment

**DOI:** 10.3390/s20071919

**Published:** 2020-03-30

**Authors:** Federico Carotenuto, Lorenzo Brilli, Beniamino Gioli, Giovanni Gualtieri, Carolina Vagnoli, Mauro Mazzola, Angelo Pietro Viola, Vito Vitale, Mirko Severi, Rita Traversi, Alessandro Zaldei

**Affiliations:** 1 Institute of BioEconomy, National Research Council of Italy (CNR IBE), 50019 Sesto Fiorentino (FI), Italy; lorenzo.brilli@ibe.cnr.it (L.B.); giovanni.gualtieri@ibe.cnr.it (G.G.); carolina.vagnoli@ibe.cnr.it (C.V.); alessandro.zaldei@ibe.cnr.it (A.Z.); 2 Institute of Polar Sciences, National Research Council of Italy (CNR ISP), 40129 Bologna (BO), Italy; mauro.mazzola@cnr.it (M.M.); angelopietro.viola@cnr.it (A.P.V.); vito.vitale@cnr.it (V.V.); 3 Chemistry Department, University of Florence, 50019 Sesto Fiorentino (FI), Italy; mirko.severi@unifi.it (M.S.); rita.traversi@unifi.it (R.T.)

**Keywords:** low-cost sensors, Arctic environment, atmospheric composition

## Abstract

The Arctic is an important natural laboratory that is extremely sensitive to climatic changes and its monitoring is, therefore, of great importance. Due to the environmental extremes it is often hard to deploy sensors and observations are limited to a few sparse observation points limiting the spatial and temporal coverage of the Arctic measurement. Given these constraints the possibility of deploying a rugged network of low-cost sensors remains an interesting and convenient option. The present work validates for the first time a low-cost sensor array (AIRQino) for monitoring basic meteorological parameters and atmospheric composition in the Arctic (air temperature, relative humidity, particulate matter, and CO_2_). AIRQino was deployed for one year in the Svalbard archipelago and its outputs compared with reference sensors. Results show good agreement with the reference meteorological parameters (air temperature (T) and relative humidity (RH)) with correlation coefficients above 0.8 and small absolute errors (≈1 °C for temperature and ≈6% for RH). Particulate matter (PM) low-cost sensors show a good linearity (r^2^ ≈ 0.8) and small absolute errors for both PM_2.5_ and PM_10_ (≈1 µg m^−3^ for PM_2.5_ and ≈3 µg m^−3^ for PM_10_), while overall accuracy is impacted both by the unknown composition of the local aerosol, and by high humidity conditions likely generating hygroscopic effects. CO_2_ exhibits a satisfying agreement with r^2^ around 0.70 and an absolute error of ≈23 mg m^−3^. Overall these results, coupled with an excellent data coverage and scarce need of maintenance make the AIRQino or similar devices integrations an interesting tool for future extended sensor networks also in the Arctic environment.

## 1. Introduction

The impact of climate change in the Arctic poses great environmental concern since temperature in Polar Regions is rising faster than in lower latitude areas [1,2,3,4], with increasing occurrence of warming events [5]. The faster warming at the poles is primarily due to the ice-albedo positive feedback, where a temperature increase induces melting of ice caps, glaciers, and sea ice, thus, reducing surface albedo and increasing surface temperature of the region [3,6,7]. Another important positive feedback due to warming is the permafrost reduction and melting, with associated greenhouse gas (GHG) emissions to the atmosphere [8]. The sensitivity of this environment may represent a tipping point for the global climate, making it an extremely important natural laboratory that requires appropriate monitoring. 

Experimental campaigns in the Artic regions are often deployed in the summer, with relatively warm temperatures and high biospheric activity. Nevertheless, year-round measurements extended to the winter season have been demonstrated to be important to fill knowledge gaps [9,10] and specific instrumental setup have been developed and assessed for Polar Regions winter measurements [11].

Given the extreme environmental conditions of the region, few atmospheric and meteorological high-quality data are available. Since single point observations are unable to capture climate dynamics at a wider spatial scale, multiple polar stations would be needed. However, the high cost for observatories building and maintenance, and the low accessibility to the area pose serious challenges to increasing the number of observation points.

In this context, the possibility of deploying low-cost sensors as auxiliary observations integrating high-cost reference observatories may result highly interesting and convenient. The deployment of low-cost sensor networks for atmospheric chemistry composition (especially air pollution) has recently known a relatively large diffusion as showed by scientific literature [12,13,14,15] and international actions (such as the European Cooperation in Science and Technology (COST) action EuNetAir) boosting innovation and technology development. The reduced cost, power consumption, and low maintenance needs make them useful tools under several environmental conditions [13,16]. In the Arctic, there are still very few cases where these kinds of sensors have been deployed for specific studies on oceans [17], animals [18], and greenhouse gas emissions from soils [19]. Despite the fact that low-cost sensors can provide useful information, the lower sensitivity compared with high-cost references make them less able to capture small variations in atmospheric parameters. Assessment studies on the behavior and performance of low-cost atmospheric sensors in harsh environment combined with low atmospheric concentrations are lacking, and needed to instruct the deployment of measurement sensor networks. Based on such assessment, the trade-off between increasing the number of measurement points (e.g. more points at lower accuracy) or increasing the sensor accuracy in a network (e.g. less points at higher accuracy), at the same total cost, could be better optimized.

Here, we presented the results achieved from the first long-term deployment of low-cost sensors to measure atmospheric parameters, namely air temperature and humidity, size resolved particles, and CO_2_ concentration. 

In this work, we: (i) evaluated the performances of the low-cost sensor array AIRQino against reference measurements from high-quality instrumentation and (ii) assessed the suitability and endurance of the low-cost sensors at detecting atmospheric and meteorological data in the Arctic environment. 

## 2. Materials and Methods

### 2.1. Study Area and Reference Stations

**Figure 1:**
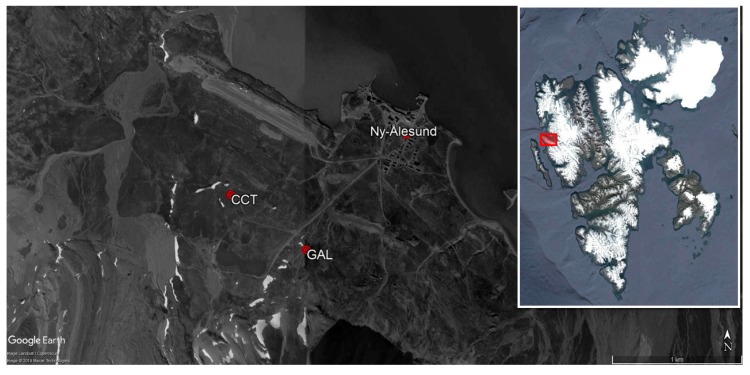
Map of the sampling area. The red marks indicate the location of the Climate Change Tower (CCT), the Gruvebadet Atmospheric Laboratory (GAL), and the Ny-Ålesund research village. The inset shows the Svalbard archipelago with a red rectangle highlighting the Brogger peninsula and the Kongsfjorden where the sampling stations are located.

Data from both the low cost and the reference stations used in this work were acquired in the Brogger Peninsula and the Kongsfjord, in the Svalbard archipelago (Figure 1). Following the Köppen–Geiger climate classification, Svalbard are considered ET (Polar-Tundra) with average temperature of warmest month between 0 and 10 °C [20]. Climate normals (1961–1990) for Ny- Ålesund (obtained from the Norwegian Meteorological Institute, www.eklima.met.no) show a minimum monthly average temperature in February (−14.6 °C) and a maximum in July (4.9 °C). Minimum average monthly precipitation is in May and June (16.5 mm) and the maximum in September (44 mm).

Two reference stations are used in this study: 

(1) The Amundsen-Nobile Climate Change Tower (CCT) is a 34 m high tower located about 1 km west of the Ny-Ålesund Research Station. It is equipped with conventional meteorological and micro-meteorological instruments, radiometers, and sensors for snow and soil measurements and it is focused to the characterization of the lower troposphere processes [21]. 

(2) The Gruvebadet Atmospheric Laboratory (GAL) is an instrumented shelter about 1 km south of Ny-Ålesund. On the basis of prevailing winds, its position allows to avoid or minimize contamination from anthropogenic sources. The main aim of this laboratory is, in fact, the monitoring of background atmospheric composition, mainly aerosols [22].

### 2.2. AIRQino Low-Cost Sensor

**Figure 2:**
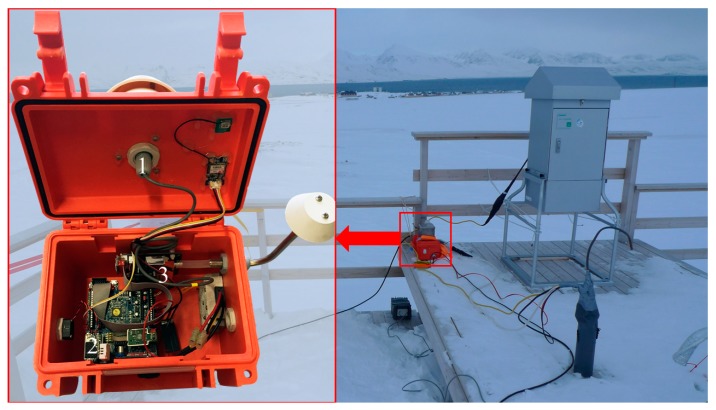
Positioning of the low-cost sensor array (AIRQino) on the roof of the Gruvebadet Atmospheric Laboratory (GAL) and interiors of the rugged enclosure highlighting the temperature (T) and relative humidity (RH) sensor (**1**), the CO_2_ sensor (**2**), and the particulate matter (PM) sensor (**3**). The snow protection cage is visible in the small red box on the right on top of the AIRQino.

The low-cost station AIRQino is a custom printed circuit board (PCB) developed by the Institute of Bioeconomy (IBE) of the National Research Council of Italy (CNR) that is able to integrate a set of low-cost sensors and transmit their data to a standard Arduino microcontroller by acting as an Arduino shield [23]. The board integrates sensors for air temperature (T) and relative humidity (RH) (AM2315 Adafruit, New York City, NY, USA), and a set of atmospheric components sensing elements including CO_2_ (S8, SenseAir, Delsbo, Sweden) and particulate matter (PM_2.5_ and PM_10_ (SDS011, Nova Fitness, Jinan, China). The sensor board, the microcontroller unit, the data storage and the sensing elements are enclosed in a rugged, waterproof enclosure. Air circulation is obtained with two Ingress protection (IP) 33 ventilation devices (mod. 3540631, Fibox Inc., Glen Burnie, MD, USA) and a MC20080V1 brushless fan (Sunon Inc., Brea, CA, USA) with a nominal flow-rate of 2.7 m^3^ h^−1^ (Figure 2). The CO_2_ sensor returns mixing ratio of the gas in ppm. The PM laser-scattering optical particle counter has a separate inlet and an internal fan able to provide continuous air recirculation at 0.75 m^3^ h^−1^ and return densities in (µg m^−3^). The only but relevant practical difference with the standard AIRQino described in [23] is the addition of a 5 W ceramic heating element and a metallic cage for snow protection of the T and RH external sensor, aimed at increasing the performance in harsh environments. A small metallic net was added around the inlet for the PM sensor to provide protection from the snow to the particulate matter sensor as well. Also, the AIRQino box was insulated with a single layer of a ceramic tissue for increased protection to thermal stresses. A low-cost GPS module was included to timestamp data in coordinated universal time (UTC) format. Data from the sensor board were sent every 5 seconds to a laptop via a serial RS-232 interface and saved as daily text files. During the sampling the AIRQino was powered at 12V DC with an AC–DC transformer connected directly to the 220V AC grid power of the GAL.

### 2.3. Reference Sensors at the CCT and GAL

The CCT mounts multiple “slow” (1 min. acquisition rate) instruments at various levels, between 2 and 33 m, including Vaisala HMP45AC (Vaisala Corporation, Helsinki, Finland) thermo-hygrometers for air temperature and relative humidity. At a 20-m height the tower also hosts a “fast” (20 Hz acquisition rate) Campbell-Scientific EC150 eddy-covariance system for measuring fluxes of water vapor and CO_2_ (Campbell-Scientific Inc., UT, USA). The temperature and relative humidity at 2 m from the ground, as well as the CO_2_ values were used for comparison with the AIRQino. A full list of the CCT sensors can be found in [21]. 

The GAL is equipped with various aerosol sampling equipment [22,24] and in particular a scanning mobility particle sizer (SMPS) model TSI 3034 and an aerodynamic particle sizer (APS) model TSI 3321 (TSI incorporated, MN, USA). The 54 channel SMPS detects nanometric particles with electrical mobility diameter between 10 and 487 nm, while the 52 channels APS detects micrometric particles with an aerodynamic equivalent diameter between 523 nm and 20.54 µm [24,25]. The two instruments are connected to a common inlet following the European Supersites for Atmospheric Aerosol Research-European Research Infrastructure for the observation of Aerosol, Clouds and Trace Gases (EUSAAR-ACTRIS) protocol and measure aerosol size distribution [25]. PM_10_ aerosol was also collected by a TECORA Skypost inertial sampler on 47 mm diameter polytetrafluoroethylene (PTFE) filters at daily resolution. Both collection of PM_10_ on filters and determination of PM_10_ mass were accomplished according to the European Standard procedure EN12341:2014 guideline (Ambient air—Standard gravimetric measurement method for the determination of the PM_10_ or PM_2.5_ mass concentration of suspended particulate matter). The sampler operated at a nominal flow rate of 2.30 m^3^ h^−1^, over a nominal sampling period of 24 h. PM_10_ was then determined by gravimetric analysis. Measurement results were expressed as densities (µg m^−3^), where the air volume is that at ambient conditions near the inlet at the time of sampling. The gravimetric method represents the reference method for measuring PM concentrations, since there are no standardized techniques for near real-time observations. PM gravimetric samplers can yield accurate measurements, considering a 10% variation of mean concentration [26].

GAL and Gravimetric PM_10_ measurements from GAL (GAL-GRAV) were not operational during the winter resulting in a lack of data between 2 October, 2017 and 21 February, 2018 for GAL and between 25 September, 2017 and 23 February, 2018 for GAL-GRAV.

### 2.4. Data Processing

All raw data from AIRQino, CCT, and GAL were de-spiked following an interquartile range-based algorithm. Outliers were defined when values were below (above) the first (third) quartile minus (plus) three times the interquartile range (i.e. the range between the first and the third quartile of the whole time series). For the reference sensors (CCT and GAL), given the higher confidence in the measurements, the small gaps generated by the de-spiking procedure were filled via linear interpolation to maximize data coverage. In addition, CO_2_ concentration from the CCT was further smoothed by a Gaussian filter with a 20 data-points window to reduce noise.

After de-spiking, all atmospheric (PM and CO_2_) and meteorological (air temperature and RH) data from AIRQino, GAL, and CCT were averaged at daily time resolution. 

AIRQino CO_2_ measurements (in ppm) were converted to density (in mg m^−3^) to be compared with the CCT, using air temperature and pressure (P) derived from the CCT itself. 

Differential logarithmic aerosol volume size distributions from GAL (dV/dlogD, in µm^3^ cm^−3^) were multiplied by a density of 1.5 g cm^−3^ following previous studies in the Boreal environment [27,28,29] and summed over the appropriate diameter range to obtain mass densities (in µg m^−3^) that are comparable with the AIRQino output. The density correction of the volume size distributions is explained by the following dimensional analysis (1):

(1) $$\frac{g}{cm^{3}}\cdot \frac{\mu m^{3}}{cm^{3}}=\frac{g}{10^{-6}m^{3}}\cdot \frac{10^{-18}m^{3}}{10^{-6}m^{3}}=\frac{g\cdot 10^{-6}}{m^{3}}=\frac{\mu g}{m^{3}}$$

Gravimetric PM10 measurements from GAL were natively referred to 24 hours intervals, therefore, no further processing was made.

To evaluate the effect of RH fluctuations on PM concentrations, root mean square errors (RMSEs) between AIRQino and GAL PM were computed on hourly data over 5%-wide RH bins.

Basic descriptive statistics, bias and normalized bias were calculated between the AIRQino and the reference sensors to characterize the performance and accuracy. Bias was calculated as:

(2) $$Bias=\frac{\sum_{i=1}^{n}\left(A_{i}-R_{i}\right)}{n}$$

where *A* is the AIRQino recorded values, *R* is the reference instrument values, and *n* the size of the sample (i.e.: the total number of matching daily observations during the measurement period). Bias is normalized on the basis of the averages of the two samples in order to obtain a percentage of over (under) estimation as:

(3) $$Normalized\;Bias=\left(\frac{Bias}{\sqrt{\bar{A}\cdot \bar{R}}}\right)\cdot 100$$

## 3. Results

**Figure 3:**
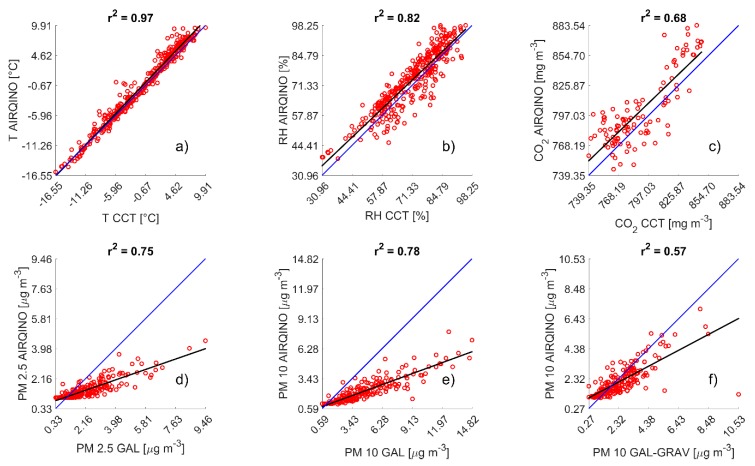
Scatterplots of AIRQino daily-averaged data versus reference sensor data (either CCT, GAL, or GAL-GRAV). Black solid lines show the lines of fit, while blue solid lines the 1:1 reference line. Each subplot is referred to a different variable: T (**a**), RH (**b**), CO_2_ (**c**), PM_2.5_ (**d**), PM_10_ (**e**), and gravimetric PM_10_ (**f**). The title of each subplot shows the calculated r^2^.

**Figure 4:**
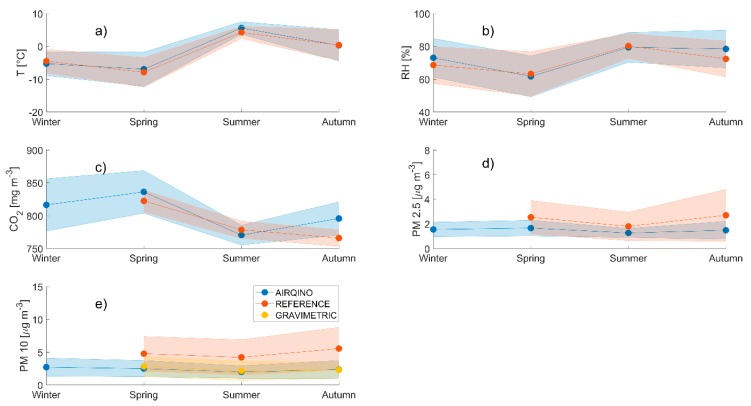
Seasonal trends of air temperature (**a**), relative humidity (**b**), CO_2_ (**c**), PM_2.5_ (**d**), and PM_10_ (**e**) for AIRQino (blue), CCT/GAL (orange), and GAL-GRAV (yellow). Shaded areas represent standard deviation.

Air temperature measured by the AIRQino showed a very good agreement with the CCT reference sensor (r^2^ = 0.97, RMSE = 1.17 °C, Figure 3a). The AIRQino exhibited overall a small overestimation (bias of 0.48 °C, normalized bias 0.18%). Seasonal temperature trends were closely followed by both AIRQino and CCT (Figure 4a) with highest values in summer (5.61 ± 1.90 °C for AIRQino and 4.26 ± 1.85 °C for CCT) and lowest in spring (−6.96 ± 5.20 °C for AIRQino and −7.89 ± 4.50 °C for CCT). Relative humidity was also in good agreement between AIRQino and CCT (r^2^ = 0.82, RMSE = 6.04 %, Figure 3b) and the seasonal trend closely mirrored air temperature (Figure 4b). Also in this case AIRQino slightly overestimated the reference sensor (bias of 2.32% and normalized bias of 3.21%) as it can be observed by the positive intercept of the linear correlation. 

Highest seasonal relative humidity was in summer (79.41 ± 9.04 % for AIRQino and 80.27 ± 7.59% for CCT) and lowest in spring (61.69 ± 12.45% for AIRQino and 63.23 ± 13.50% for CCT). 

CO_2_ data showed a significant agreement between AIRQino and CCT albeit to a lesser degree compared with meteorological data (r^2^ = 0.68 RMSE = 23.13 mg m^−3^, Figure 3c). The AIRQino overestimated the reference sensor by 12.15 mg m^−3^, but when the bias is normalized on the sensors’ averages this resulted in only a small relative overestimation (normalized bias of 1.53%). Seasonal trends for AIRQino exhibited a minimum in summer (770.29 ± 15.04 mg m^−3^) and a maximum in spring (836.20 ± 32.45 mg m^−3^), while the minimum for CCT was in autumn (765.84 ± 12.63 mg m^−3^) and the maximum for the CCT was also in spring (822.41 ± 16.62 mg m^−3^), while incomplete data coverage didn’t allow to assess an average winter concentration. 

PM_2.5_ showed a relatively high correlation between AIRQino and GAL (r^2^ = 0.75, RMSE = 1.27 µg m^−3^, Figure 3d) but a consistent underestimation by the AIRQino (Figure 4d, bias of -0.77 µg m^−3^, and normalized bias of −42.40%). 

Similarly, AIRQino and GAL PM_10_ trends were in agreement (r^2^ = 0.78, RMSE = 3.06 µg m^−3^, Figure 3e), but AIRQino was generally underestimating PM_10_ concentrations (Figure 4e, bias of −2.40 µg m^−3^ and normalized bias of −73.42%). 

AIRQino and GAL-GRAV PM_10_ trend data also showed a relatively good agreement, albeit lower than with the optical instrument (r^2^ = 0.57, RMSE = 1.04 µg m^−3^, Figure 3f). AIRQino concentrations were closer to gravimetric data than to the optical one, yielding lower errors and a slight underestimation (bias = −0.31 µg m^−3^ and normalized bias = −13.24%). 

Neither PM_2.5_ nor PM_10_ showed a marked seasonal trend even if it must be considered that incomplete data coverage didn’t allow to evaluate an average winter value for the reference instruments.

**Figure 5:**
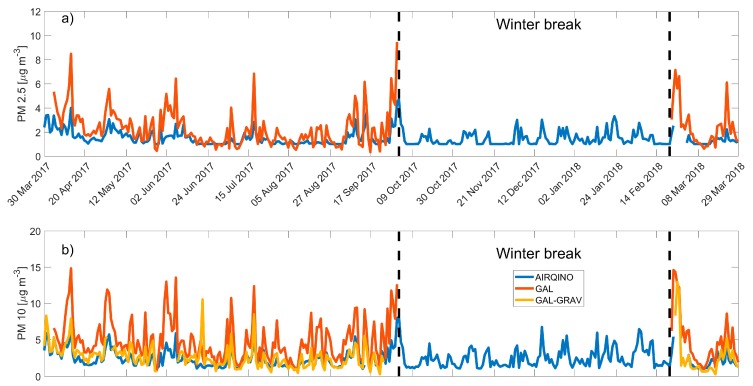
Time trends of PM_2.5_ (**a**) and PM_10_ (**b**) for AIRQino sensor (blue), GAL (orange line), and GAL-GRAV (yellow line). Dashed black lines delimitate the winter period of inactivity of the GAL.

Time series of daily averages of particulate matter were in agreement between AIRQino, GAL and GAL-GRAV when all the devices were operational (Figure 5a,b). 

**Figure 6:**
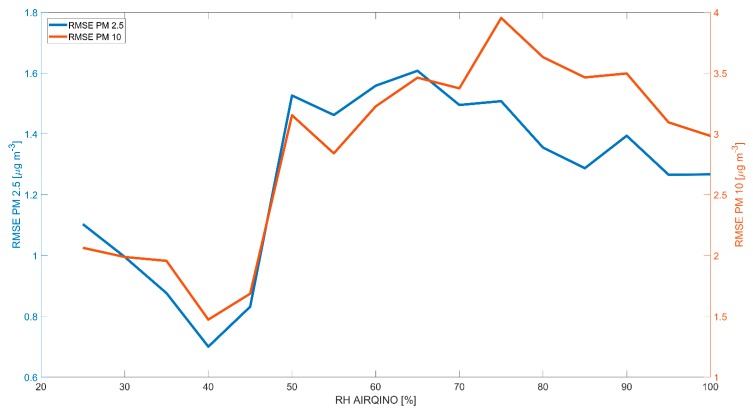
Root mean square errors (RMSE) between the AIRQino PM_2.5_ (blue line, left y-axis), AIRQino PM_10_ (orange line, right *y*-axis), and GAL. Hourly PM data were binned on the basis of 5%-wide RH bins (*x*-axis) and RMSE was computed over such bins.

Independently from seasonal patterns, the RMSE between AIRQino and GAL particulate matter showed a dependence from RH as it’s seen from Figure 6. 

Table 1 summarizes the results for the whole year of comparison of daily averages of AIRQino and reference sensors for the various variables.

## 4. Discussion

The AIRQino meteorological sensor showed an overall agreement with the reference sensors both in terms of trends and magnitudes. Air temperature in Svalbard is known to increase in summer well above zero, and even if less intensively, relative humidity is also known to increase in the warmer months [30] due to a general acceleration of the water cycle. Seasonal fluctuations in computed r^2^ and RMSE for AIRQino T and RH were present, but they did not impact the overall sensor performances (Table 1) and were not due to sensor drift effects (as seen by computing the same statistics over a 10-days sliding window). CO_2_ emissions in the Arctic environment depend on snowmelt and soil and vegetation respiration [31], with a net CO_2_ absorption in the summer rather than in winter [9]. While CO_2_ concentrations are not a direct measurement of the emissions, the two entities are linked (given that surface flux contributes to drive atmospheric concentration at large spatial scales, see [32]) and the AIRQino, in fact, showed higher average seasonal concentrations in winter compared with summer. Overall, the AIRQino CO_2_ sensor tended to be closer to the CCT in the lowest values and overestimate higher ones (Table 1). This could also explain the seasonal differences seen in autumn in Figure 4c, where the CCT detects a seasonal minimum that is not detected by the AIRQino. Given the long stretches of missing data due to the CCT datalogger failures, though, it is hard to discuss if this is actually a shortcoming of the low-cost sensor itself rather than an effect of the data shortage. In order to compare AIRQino and GAL PM concentrations it was necessary to assign a particle density to the GAL data in order to derive a mass concentration from an aerosol volume size distribution. The density value was assigned following the available literature reporting particulate matter density in boreal areas, but studies done on airborne particulate matter in Svalbard show a large variability in the composition of PM in all size ranges [24,33]. This difference in composition, arisen from a high variability in dominant sources as function of the season [22,34] and ranging between chlorides to metal oxides and other species [33], would suggest that particles of different densities have been measured by the GAL and also by AIRQino. Overall, this is a limitation of optical methods that is worth to address here: unknown chemical composition, or variable composition, pose challenges to the conversion from optical to quantitative variables, and this applies to both GAL and AIRQino. Assuming that the chosen density is representative for the Arctic environment, though, the AIRQino was able to capture the day-to-day variability of both PM_2.5_ and PM_10_, as it is confirmed by the high r^2^ values (0.75 and 0.78, respectively), low RMSE (1.27 and 3.06 µg m^−3^, respectively) and the low bias (−0.77 and −2.40 µg m^−3^, respectively). Gravimetric concentrations (GAL-GRAV) are generally lower than those measured optically (GAL). In fact, while the trends between PM_10_ GAL and PM_10_ GAL-GRAV are similar, with an r^2^ of 0.65 (*p* < 0.05) and an RMSE of 2.48 µg m^−3^, the GAL tends to overestimate the gravimetric concentration (normalized bias = 56.51 %). This suggests that the literature reported density for aerosol in the Arctic environment is in the ballpark, but not exactly the one found in Ny-Ålesund. In fact, by comparing AIRQino and GAL with GAL-GRAV it is possible to derive approximate particle density at least for PM_10_. Fitting a zero-intercept linear model between AIRQino and GAL-GRAV yielded that AIRQino measurements should be corrected by a factor of 1.13 in order to match gravimetric ones. This factor is not a real density given that it takes into account both the between-sensors differences and the actual analog-to-digital conversion in the SDS011 between laser scattering and output measurement, but since it is close to 1, it suggests that the SDS011 is quite close to the actual particle density. Fitting a similar model to the aerosol volume size distributions from GAL (dV/dlogD, in µm^3^ cm^−3^), instead, yielded an effective density for PM_10_ of 0.85 g cm^−3^. This is in accordance with the results of Table 1 which shows how the GAL (using the literature density of 1.5 g cm^−3^) returned higher values when compared with AIRQino and GAL-GRAV. It is clear that more research is needed to address this topic in the Arctic environment, especially in view of deploying a low-cost sensor network in the future.

Even if this is the first evaluation in the Arctic environment, the SDS011 sensor was tested in the northern city of Oslo (Norway) and showed similarly high correlations coefficients with a reference sensor for PM_2.5_ ranging from 0.55 to 0.71, an RMSE < 6 µg m^−3^ and accuracies >80% [35]. The good linearity of the SDS011 sensor for PM_2.5_ integrated in the AIRQino were evaluated both in the field and in the laboratory: in the field correlations of up to 0.9 where found with reference sensors when the SDS011 was deployed in different urban environments such as Thessaloniki (Greece) and Wrocław (Poland) [36,37]. Similarly, high coefficients (>0.9) were also found in the laboratory when the sensor was tested in an aerosol chamber [38]. The AIRQino itself was tested in a similarly temperate urban environment in Florence (Italy) obtaining raw correlation coefficients >0.8 when compared with reference stations [23]. Nevertheless, in the Arctic deployment, while the SDS011 showed low absolute errors, the high negative relative bias (>−40%) demonstrated a consistent underestimation of the reference PM that wasn’t seen, for example, in the deployment of the AIRQino in Florence (where the normalized mean bias was around 5% for both PM_2.5_ and PM_10_, [23]). The main difference between the Arctic deployment and the other literature studies on SDS011 is the dynamic range of PM experienced by the sensor and the aerosol chemical composition. While in urban contexts the sensor experiences a range of PM from close to zero up to tens of micrograms per cubic meter (even above 100 µg m^−3^ in [37], while [39] have found PM_10_ concentrations around 40 µg m^−3^ in urban sites in Tuscany), in Ny-Ålesund the range measured by the GAL (GAL-GRAV) does not go above 15 (13) µg m^−3^ for PM_10_ and not above 10 µg m^−3^ for PM_2.5_. In fact, [37] saw increases in relative errors when PM_2.5_ concentrations were below or equal 20 µg m^−3^. Another factor affecting the accuracy of the SDS011 is humidity resulting in a drop in performance (i.e.: increased RMSE) at increasing RH for both PM_2.5_ and PM_10_, especially at values >50% (Figure 6). This is likely related to hygroscopic effects: as average RH increase, the local occurrence of condensation of small water droplets in the inlet system also increases, generating particles of different size distribution and optical properties with respect to the aerosol of that air parcel, impacting significantly the measurement accuracy. In fact, [40] noted a humidity-related scattering enhancement that can go up to 3.14 for Arctic aerosols. Similar effects are also reported in the literature for the same sensor: both [35] and [37] also shown an increased discrepancy between the SDS011 and reference sensor for PM_2.5_ when RH increased above 80%. Reduced correlations between reference and low-cost sensor at high humidity were also seen by [41] on PM_2.5_ during the deployment of a similar low-cost sensor (SDS019, Nova Fitness, Jinan, China) on a high-elevation mountain. 

During the one year of deployment, the AIRQino sensor dropped only 0.19 % of the transmitted data packages and kept running without the necessity of any maintenance, proving to be well-suited to work in polar environments. While in the present application the data were streamed to a PC via a serial interface, AIRQino has the capability to integrate a wide range of wireless connectivity options (e.g., cellular, Bluetooth, and WiFi) and also an SD card reader to work in a standalone mode even when no kind of connectivity is present or radio communications are limited due to frequency restrictions (such as in Ny-Ålesund, for example, http://nysmac.npolar.no/practical/radio-silent-area.html). In this configuration, data are written to a comma delimited text file and, due to the small size of each individual data message (roughly 180 bytes), they can be accumulated for long periods of time (10.6 years considering one message per minute and a 128 GB SD card). Deploying the AIRQino on the roof of the GAL observatory allowed to have a continuous supply of power from the grid, but a deployment of a sensor network in fully remote areas would face the issue of powering up the sensors without grid access. The AIRQino power draw without the heating element is of 200 mAh^−1^ @ 12 VDC of which 150 mAh^−1^ @ 5 VDC are used by the sensor array. The total consumption of the AIRQino is ≈ 2.5 Wh^−1^, 30% of which is used by the sensor array [23]. Given the modest power draw, the sensor could be powered through a relatively small solar panel (between 10–25 W) in boreal areas where Sun is consistently present during summer. During the polar night, the AIRQino could receive power with different solutions such as small wind turbines in combination with lithium-ion batteries or fuel cells [42,43]. Another possibility to reduce power draw may come from the usage of newer generation of sensors designed specifically for low-powered applications [44,45]. Powering a sensor network in remote Arctic areas remains a complex logistic issue that will require further investigation and engineering innovation.

## 5. Conclusions

In this work a low-cost sensor for atmospheric composition (AIRQino) was tested in the Arctic environment for one year alongside some high-cost reference sensors. Even in such an extreme environment, with often low concentrations of certain atmospheric components (such as CO_2_ and particulate matter), the AIRQino was in general agreement with the reference sensors. The performance assessment of meteorological data (T and RH), CO_2_, PM_2.5_, and PM_10_ was presented, using long-running reference sensors in close proximity with AIRQino, but future work will need to focus on the other onboard sensors of the AIRQino (CO, O_3_, and NO_2_) in order to fully characterize the polar performances of this low-cost station. Further work would be needed also to better characterize PM_2.5_ and PM_10_ magnitude and behavior, given the lack of an in-depth study of particle density in Svalbard. In fact, the analysis of gravimetric measurements highlighted that the particle density reported by other literature studies on Arctic aerosol brings to an overestimation of the gravimetric concentration by the optical instruments. High humidity conditions were observed to decrease the particles measurement accuracy. To overcome this limitation, solutions aimed at preventing condensation in the inlet could be investigated (e.g., heating the inlet), keeping in mind the trade-off between desirable increase in accuracy and undesirable increase in power drain, system complexity, and cost. Given the proven reliability and the low power drain of the AIRQino station, deployment of a sensor network to spatially characterize atmospheric composition in even more remote Arctic areas appears feasible. With no grid power available, further energy production optimization would be required to provide AIRQino with constant power throughout the year and the polar night.

## Figures and Tables

**Table 1:** Metrics and statistics between the AIRQino and the reference sensors. For GAL-GRAV the statistics are referred to the comparison AIRQino.

Variable	Units	Instrument	Minimum	Average	Maximum	Comparison Statistics			
						r^2^ (p)	RMSE	Bias	Norm. Bias
T	°C	AIRQino	−16.22	−1.50	9.52	0.97 (<0.05)	1.17	0.48	0.18 %
		CCT	−16.55	−1.74	9.91				
RH	%	AIRQino	38.47	73.16	98.25	0.82 (<0.05)	6.04	2.32	3.21 %
		CCT	30.96	71.35	95.38				
CO_2_	mg m^−3^	AIRQino	735.85	803.44	911.29	0.68 (<0.05)	23.13	12.15	1.53 %
		CCT	739.35	789.90	847.82				
PM_2.5_	µg m^−3^	AIRQino	1.00	1.48	4.73	0.75 (<0.05)	1.27	-0.77	−42.40 %
		GAL	0.33	2.31	9.46				
PM_10_	µg m^−3^	AIRQino	1.00	2.37	8.14	0.78 (<0.05)	3.06	-2.40	−73.42 %
		GAL	0.59	4.81	14.82				
		GAL-GRAV	0.27	2.59	12.95	0.57 (<0.05)	1.04	-0.31	−13.24%
